# Cyanidotetra­kis­(trimethyl­phosphine)cobalt(I)

**DOI:** 10.1107/S160053681101083X

**Published:** 2011-03-26

**Authors:** Xiaofeng Xu, Lei Feng, Xiaoyan Li

**Affiliations:** a School of Chemistry and Chemical Engineering, Shandong University, Jinan 250100, People’s Republic of China

## Abstract

The title compound, [Co(CN)(C_3_H_9_P)_4_], was obtained as a product of the reaction of [Co(PMe_3_)_4_] with a molar equivalent of 2,6-difluoro­benzonitrile in diethyl ether. This compound is stable in the air for several hours, but rapidly decomposes at room temperature in solution. The cobalt(I) atom has s trigonal–bipyramidal coordination enviroment in which the cyano group and one of the PMe_3_ groups are in the axial positions.

## Related literature

For related cobalt(II) compounds, see: Yu *et al.* (2008 ▶); Li *et al.* (2006 ▶).
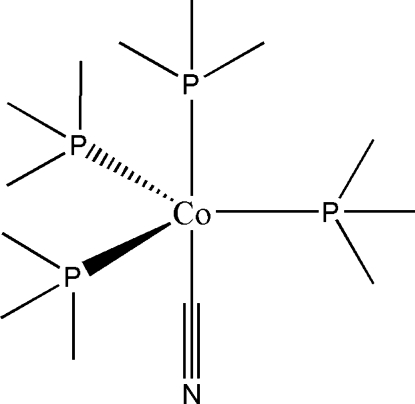


## Experimental

### 

#### Crystal data

[Co(CN)(C_3_H_9_P)_4_]
*M* *_r_* = 389.24
Monoclinic, 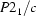

*a* = 13.160 (3) Å
*b* = 9.6136 (19) Å
*c* = 17.971 (4) Å
β = 93.09 (3)°
*V* = 2270.3 (8) Å^3^
*Z* = 4
Mo *K*α radiation
μ = 1.03 mm^−1^
*T* = 293 K
0.25 × 0.23 × 0.22 mm

#### Data collection

Bruker APEXII diffractometer
Absorption correction: multi-scan (*SADABS*; Bruker, 2001 ▶) *T*_min_ = 0.395, *T*_max_ = 0.434
11732 measured reflections
4438 independent reflections
3663 reflections with *I* > 2σ(*I*)
*R*_int_ = 0.020

#### Refinement

*R*[*F* ^2^ > 2σ(*F* ^2^)] = 0.068
*wR*(*F* ^2^) = 0.216
*S* = 1.06
4438 reflections
185 parameters
150 restraints
H-atom parameters constrained
Δρ_max_ = 1.06 e Å^−3^
Δρ_min_ = −2.00 e Å^−3^

### 

Data collection: *APEX2* (Bruker, 2004 ▶); cell refinement: *SAINT-Plus* (Bruker, 2001 ▶); data reduction: *SAINT-Plus*; program(s) used to solve structure: *SHELXS97* (Sheldrick, 2008 ▶); program(s) used to refine structure: *SHELXL97* (Sheldrick, 2008 ▶); molecular graphics: *SHELXTL* (Sheldrick, 2008 ▶); software used to prepare material for publication: *SHELXTL*.

## Supplementary Material

Crystal structure: contains datablocks global, I. DOI: 10.1107/S160053681101083X/om2401sup1.cif

Structure factors: contains datablocks I. DOI: 10.1107/S160053681101083X/om2401Isup2.hkl

Additional supplementary materials:  crystallographic information; 3D view; checkCIF report

## Figures and Tables

**Table:** 

C13—N1	1.166 (7)
Co1—C13	1.896 (5)
Co1—P2	2.2018 (15)
Co1—P1	2.2082 (17)
Co1—P4	2.2115 (17)
Co1—P3	2.2272 (17)

**Table:** 

N1—C13—Co1	178.8 (6)
C13—Co1—P2	179.02 (19)
C13—Co1—P1	83.92 (19)
P2—Co1—P1	97.05 (7)
C13—Co1—P4	83.39 (18)
P2—Co1—P4	96.24 (6)
P1—Co1—P4	119.14 (8)
C13—Co1—P3	84.66 (19)
P2—Co1—P3	94.75 (7)
P1—Co1—P3	118.05 (8)
P4—Co1—P3	119.55 (8)

**Fig. 1.**
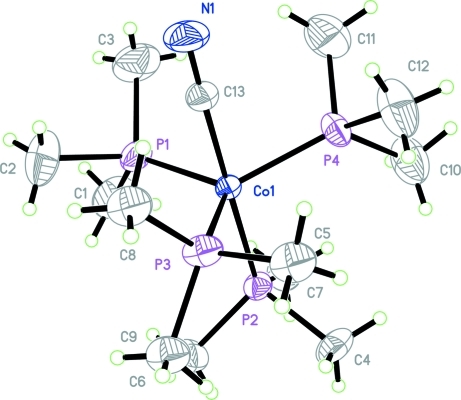
A view of the structure of the title compound showing 30% probability displacement ellipsoids.

## supplementary crystallographic information

### Comment

In the title molecule (Fig. 1, Table 1) the cobalt(I) atom has trigonal planar coordination geometry. Three PMe_3_ groups using P1, P3 and P4 form the trigonal plane and the fourth PMe_3_ group, using P2, and the cyano group are in the axial position. The axial groups are linear with P2-Co1-C13 of 179.02 (1)°. The sum of the bond angles in the trigonal plane (356.75 (8)°) indicates only slightly distorted planarity. We have previously reported two related cobalt(II) structures. In one, (Yu *et al.*, 2008), the axial PMe_3_ group is replaced by a phenyl and in the other, it is replaced by a 2,6-difluorophenyl group (Li *et al.*, 2006). These structures show a distorted square pyramidal coordination, which is different from the title compound.

### Experimental

A sample of Co(PMe_3_)_4_ (1.0 g, 2.75 mmol) in 30 ml of diethyl ether was combined with a solution of 2,6-difluorobenzonitrile (0.19 g, 1.36 mmol) in diethyl ether (20 ml) at -80%A. The reaction mixture was warmed to ambient temperature and stirred for 24 h to form a red solution. The volatiles were removed *in vacuo*, and the resulting solid was extracted with pentane (50 ml). Crystallization from its pentane solution at -15 °C afforded red crystals suitable for X-ray diffraction analysis (yield 0.22 g, 42%), dec > 120 °C. The datum crystal was coated with perfluoropolyether to retard decomposition due to air sensitivity during data collection.

### Refinement

All H atoms on C were placed in calculated positions with a C—H bond distance of 0.96 Å and *U*_iso_(H) = 1.5*U*_eq_ of the carrier atom. Large thermal motion of the methyl groups required that restraints be applied to the thermal parameters of several atoms, namely, SIMU 0.001 0.002 3.8 P3 C7 C8 C9; SIMU 0.01 0.02 3.8 P4 C10 C11 C12; ISOR 0.01 $C. The maximum and minimum peaks in the final electron density map had values of 1.06 e/Å^3^ and -2.00 e/Å^3^ which were located 0.03 Å from P3 and 0.05 Å from C7, respectively.

### Crystal data

**Table:**

[Co(CN)(C_3_H_9_P)_4_]	*F*(000) = 832.0
*M**_r_* = 389.24	*D*_x_ = 1.139 Mg m^−^^3^
Monoclinic, *P*2_1_/*c*	Mo *K*α radiation, λ = 0.71073 Å
Hall symbol: -P 2ybc	Cell parameters from 4976 reflections
*a* = 13.160 (3) Å	θ = 2.3–26.0°
*b* = 9.6136 (19) Å	µ = 1.03 mm^−^^1^
*c* = 17.971 (4) Å	*T* = 293 K
β = 93.09 (3)°	Block, red
*V* = 2270.3 (8) Å^3^	0.25 × 0.23 × 0.22 mm
*Z* = 4	

### Data collection

**Table:**

Bruker APEXII diffractometer	4438 independent reflections
Radiation source: fine-focus sealed tube	3663 reflections with *I* > 2σ(*I*)
graphite	*R*_int_ = 0.020
φ and ω scans	θ_max_ = 26.0°, θ_min_ = 2.3°
Absorption correction: multi-scan (*SADABS*; Bruker, 2001)	*h* = −15→16
*T*_min_ = 0.395, *T*_max_ = 0.434	*k* = −11→11
11732 measured reflections	*l* = −15→22

### Refinement

**Table:**

Refinement on *F*^2^	Primary atom site location: structure-invariant direct methods
Least-squares matrix: full	Secondary atom site location: difference Fourier map
*R*[*F*^2^ > 2σ(*F*^2^)] = 0.068	Hydrogen site location: inferred from neighbouring sites
*wR*(*F*^2^) = 0.216	H-atom parameters constrained
*S* = 1.06	*w* = 1/[σ^2^(*F*_o_^2^) + (0.1278*P*)^2^ + 4.380*P*] where *P* = (*F*_o_^2^ + 2*F*_c_^2^)/3
4438 reflections	(Δ/σ)_max_ < 0.001
185 parameters	Δρ_max_ = 1.06 e Å^−^^3^
150 restraints	Δρ_min_ = −2.00 e Å^−^^3^

### Special details

**Table:**

Geometry. All e.s.d.'s (except the e.s.d. in the dihedral angle between two l.s. planes) are estimated using the full covariance matrix. The cell e.s.d.'s are taken into account individually in the estimation of e.s.d.'s in distances, angles and torsion angles; correlations between e.s.d.'s in cell parameters are only used when they are defined by crystal symmetry. An approximate (isotropic) treatment of cell e.s.d.'s is used for estimating e.s.d.'s involving l.s. planes.
Refinement. Refinement of *F*^2^ against ALL reflections. The weighted *R*-factor *wR* and goodness of fit *S* are based on *F*^2^, conventional *R*-factors *R* are based on *F*, with *F* set to zero for negative *F*^2^. The threshold expression of *F*^2^ > σ(*F*^2^) is used only for calculating *R*-factors(gt) *etc*. and is not relevant to the choice of reflections for refinement. *R*-factors based on *F*^2^ are statistically about twice as large as those based on *F*, and *R*- factors based on ALL data will be even larger.

### Fractional atomic coordinates and isotropic or equivalent isotropic displacement parameters (Å^2^)

**Table:**

	*x*	*y*	*z*	*U*_iso_*/*U*_eq_	
C1	0.0427 (7)	0.5719 (11)	0.6108 (6)	0.112 (3)	
H1A	−0.0142	0.5545	0.6407	0.167*	
H1B	0.0756	0.4855	0.6002	0.167*	
H1C	0.0194	0.6154	0.5649	0.167*	
C2	0.1727 (9)	0.5681 (11)	0.7382 (6)	0.125 (4)	
H2A	0.2114	0.6174	0.7766	0.187*	
H2B	0.2134	0.4948	0.7192	0.187*	
H2C	0.1130	0.5292	0.7585	0.187*	
C3	0.0419 (8)	0.7949 (12)	0.7113 (7)	0.123 (4)	
H3A	−0.0118	0.7361	0.7271	0.184*	
H3B	0.0140	0.8651	0.6782	0.184*	
H3C	0.0754	0.8383	0.7540	0.184*	
C4	0.3468 (6)	0.7624 (9)	0.4375 (4)	0.080 (2)	
H4A	0.4136	0.7418	0.4588	0.120*	
H4B	0.3416	0.8603	0.4273	0.120*	
H4C	0.3353	0.7112	0.3920	0.120*	
C5	0.1325 (6)	0.7398 (9)	0.4449 (4)	0.0768 (19)	
H5A	0.1354	0.6858	0.4002	0.115*	
H5B	0.1263	0.8366	0.4323	0.115*	
H5C	0.0748	0.7115	0.4716	0.115*	
C6	0.2570 (7)	0.5211 (8)	0.4942 (4)	0.080 (2)	
H6A	0.1998	0.4794	0.5167	0.120*	
H6B	0.3189	0.4877	0.5186	0.120*	
H6C	0.2557	0.4971	0.4424	0.120*	
C7	0.5231 (5)	0.8559 (9)	0.6188 (4)	0.0783 (9)	
H7A	0.5086	0.8925	0.5697	0.117*	
H7B	0.5871	0.8078	0.6203	0.117*	
H7C	0.5264	0.9309	0.6541	0.117*	
C8	0.4623 (6)	0.7086 (8)	0.7401 (4)	0.0771 (9)	
H8A	0.4308	0.6273	0.7597	0.116*	
H8B	0.4438	0.7887	0.7682	0.116*	
H8C	0.5349	0.6975	0.7436	0.116*	
C9	0.4766 (5)	0.5759 (8)	0.6060 (4)	0.0770 (9)	
H9A	0.5466	0.5704	0.6237	0.115*	
H9B	0.4727	0.5797	0.5525	0.115*	
H9C	0.4407	0.4954	0.6222	0.115*	
C10	0.1996 (9)	1.0851 (10)	0.4832 (5)	0.114 (3)	
H10A	0.1993	1.1849	0.4826	0.172*	
H10B	0.1330	1.0512	0.4683	0.172*	
H10C	0.2479	1.0513	0.4493	0.172*	
C11	0.1338 (10)	1.1173 (12)	0.6267 (7)	0.134 (3)	
H11A	0.1330	1.0866	0.6775	0.201*	
H11B	0.0691	1.0984	0.6016	0.201*	
H11C	0.1470	1.2154	0.6255	0.201*	
C12	0.3430 (9)	1.1417 (11)	0.5971 (7)	0.123 (3)	
H12A	0.4025	1.1046	0.5757	0.185*	
H12B	0.3557	1.1521	0.6499	0.185*	
H12C	0.3270	1.2308	0.5754	0.185*	
C13	0.2731 (5)	0.8878 (6)	0.7120 (3)	0.0545 (13)	
Co1	0.26139 (5)	0.80567 (6)	0.61596 (3)	0.0371 (3)	
N1	0.2797 (6)	0.9405 (7)	0.7705 (3)	0.0869 (19)	
P1	0.13485 (13)	0.68895 (18)	0.66233 (9)	0.0621 (5)	
P2	0.25026 (12)	0.71209 (15)	0.50400 (7)	0.0479 (4)	
P3	0.41947 (12)	0.7316 (2)	0.64284 (9)	0.0605 (4)	
P4	0.23568 (14)	1.02279 (15)	0.57888 (9)	0.0598 (4)	

### Atomic displacement parameters (Å^2^)

**Table:**

	*U*^11^	*U*^22^	*U*^33^	*U*^12^	*U*^13^	*U*^23^
C1	0.099 (5)	0.129 (7)	0.109 (6)	−0.062 (5)	0.023 (5)	−0.026 (5)
C2	0.133 (7)	0.130 (7)	0.111 (6)	−0.039 (6)	0.015 (5)	0.045 (6)
C3	0.097 (6)	0.142 (8)	0.136 (7)	−0.010 (5)	0.061 (6)	−0.021 (6)
C4	0.091 (5)	0.105 (5)	0.046 (3)	−0.014 (4)	0.021 (3)	−0.007 (3)
C5	0.079 (4)	0.086 (4)	0.063 (4)	−0.010 (4)	−0.019 (3)	−0.003 (3)
C6	0.104 (5)	0.061 (4)	0.076 (4)	0.000 (4)	0.002 (4)	−0.019 (3)
C7	0.0674 (16)	0.0953 (17)	0.0713 (16)	0.0130 (15)	−0.0047 (14)	−0.0044 (15)
C8	0.0674 (15)	0.0951 (17)	0.0678 (16)	0.0156 (15)	−0.0053 (14)	−0.0044 (15)
C9	0.0670 (15)	0.0934 (17)	0.0696 (16)	0.0167 (15)	−0.0053 (14)	−0.0069 (15)
C10	0.149 (6)	0.085 (5)	0.106 (5)	0.007 (5)	−0.026 (5)	0.023 (4)
C11	0.153 (7)	0.102 (6)	0.147 (7)	0.044 (5)	0.013 (6)	0.008 (5)
C12	0.141 (6)	0.082 (5)	0.142 (6)	−0.031 (5)	−0.036 (5)	0.022 (5)
C13	0.063 (3)	0.056 (3)	0.044 (3)	0.002 (3)	0.003 (2)	−0.005 (2)
Co1	0.0414 (4)	0.0375 (4)	0.0323 (4)	0.0004 (2)	0.0023 (3)	−0.0018 (2)
N1	0.117 (5)	0.090 (4)	0.053 (3)	0.013 (4)	0.001 (3)	−0.026 (3)
P1	0.0630 (10)	0.0687 (10)	0.0564 (9)	−0.0182 (7)	0.0193 (7)	−0.0050 (7)
P2	0.0575 (8)	0.0509 (8)	0.0353 (7)	−0.0059 (6)	0.0012 (6)	−0.0048 (5)
P3	0.0509 (8)	0.0800 (10)	0.0498 (8)	0.0160 (7)	−0.0038 (6)	−0.0075 (7)
P4	0.0747 (10)	0.0405 (8)	0.0628 (9)	0.0027 (7)	−0.0097 (8)	0.0039 (6)

### Geometric parameters (Å, °)

**Table:**

C1—P1	1.863 (8)	C7—H7C	0.9600
C1—H1A	0.9600	C8—P3	1.820 (7)
C1—H1B	0.9600	C8—H8A	0.9600
C1—H1C	0.9600	C8—H8B	0.9600
C2—P1	1.840 (10)	C8—H8C	0.9600
C2—H2A	0.9600	C9—P3	1.816 (7)
C2—H2B	0.9600	C9—H9A	0.9600
C2—H2C	0.9600	C9—H9B	0.9600
C3—P1	1.850 (9)	C9—H9C	0.9600
C3—H3A	0.9600	C10—P4	1.858 (9)
C3—H3B	0.9600	C10—H10A	0.9600
C3—H3C	0.9600	C10—H10B	0.9600
C4—P2	1.855 (7)	C10—H10C	0.9600
C4—H4A	0.9600	C11—P4	1.866 (11)
C4—H4B	0.9600	C11—H11A	0.9600
C4—H4C	0.9600	C11—H11B	0.9600
C5—P2	1.850 (7)	C11—H11C	0.9600
C5—H5A	0.9600	C12—P4	1.833 (10)
C5—H5B	0.9600	C12—H12A	0.9600
C5—H5C	0.9600	C12—H12B	0.9600
C6—P2	1.847 (7)	C12—H12C	0.9600
C6—H6A	0.9600	C13—N1	1.166 (7)
C6—H6B	0.9600	Co1—C13	1.896 (5)
C6—H6C	0.9600	Co1—P2	2.2018 (15)
C7—P3	1.881 (8)	Co1—P1	2.2082 (17)
C7—H7A	0.9600	Co1—P4	2.2115 (17)
C7—H7B	0.9600	Co1—P3	2.2272 (17)
			
P1—C1—H1A	109.5	P4—C10—H10B	109.5
P1—C1—H1B	109.5	P4—C10—H10C	109.5
H1A—C1—H1B	109.5	P4—C11—H11A	109.5
P1—C1—H1C	109.5	P4—C11—H11B	109.5
H1A—C1—H1C	109.5	P4—C11—H11C	109.5
H1B—C1—H1C	109.5	P4—C12—H12A	109.5
P1—C2—H2A	109.5	P4—C12—H12B	109.5
P1—C2—H2B	109.5	P4—C12—H12C	109.5
H2A—C2—H2B	109.5	N1—C13—Co1	178.8 (6)
P1—C2—H2C	109.5	C13—Co1—P2	179.02 (19)
H2A—C2—H2C	109.5	C13—Co1—P1	83.92 (19)
H2B—C2—H2C	109.5	P2—Co1—P1	97.05 (7)
P1—C3—H3A	109.5	C13—Co1—P4	83.39 (18)
P1—C3—H3B	109.5	P2—Co1—P4	96.24 (6)
P1—C3—H3C	109.5	P1—Co1—P4	119.14 (8)
P2—C4—H4A	109.5	C13—Co1—P3	84.66 (19)
P2—C4—H4B	109.5	P2—Co1—P3	94.75 (7)
H4A—C4—H4B	109.5	P1—Co1—P3	118.05 (8)
P2—C4—H4C	109.5	P4—Co1—P3	119.55 (8)
H4A—C4—H4C	109.5	C2—P1—C3	98.9 (6)
H4B—C4—H4C	109.5	C2—P1—C1	97.5 (5)
P2—C5—H5A	109.5	C3—P1—C1	98.0 (5)
P2—C5—H5B	109.5	C2—P1—Co1	114.9 (3)
H5A—C5—H5B	109.5	C3—P1—Co1	115.5 (4)
P2—C5—H5C	109.5	C1—P1—Co1	127.1 (3)
H5A—C5—H5C	109.5	C6—P2—C5	97.6 (4)
H5B—C5—H5C	109.5	C6—P2—C4	99.2 (4)
P2—C6—H6A	109.5	C5—P2—C4	100.1 (4)
P2—C6—H6B	109.5	C6—P2—Co1	119.5 (3)
H6A—C6—H6B	109.5	C5—P2—Co1	118.6 (3)
P2—C6—H6C	109.5	C4—P2—Co1	117.9 (3)
H6A—C6—H6C	109.5	C9—P3—C8	97.9 (3)
H6B—C6—H6C	109.5	C9—P3—C7	96.9 (4)
P3—C7—H7A	109.5	C8—P3—C7	96.1 (4)
P3—C7—H7B	109.5	C9—P3—Co1	125.8 (2)
P3—C7—H7C	109.5	C8—P3—Co1	119.0 (2)
P3—C8—H8A	109.5	C7—P3—Co1	115.3 (2)
P3—C8—H8B	109.5	C12—P4—C10	96.9 (5)
P3—C8—H8C	109.5	C12—P4—C11	100.4 (6)
P3—C9—H9A	109.5	C10—P4—C11	96.6 (5)
P3—C9—H9B	109.5	C12—P4—Co1	115.5 (4)
P3—C9—H9C	109.5	C10—P4—Co1	127.6 (3)
P4—C10—H10A	109.5	C11—P4—Co1	115.0 (4)
